# Post-bariatric pregnancy is associated with vitamin K1 deficiency, a case control study

**DOI:** 10.1186/s12884-024-06407-0

**Published:** 2024-04-02

**Authors:** Brit Torunn Bechensteen, Cindhya Sithiravel, Ellen Marie Strøm-Roum, Heidi Kathrine Ruud, Gunnhild Kravdal, Jacob A. Winther, Tone G. Valderhaug

**Affiliations:** 1 Department of Endocrinology, Akershus University Hospital HF, Lørenskog, Norway; 2 Department of Clinical nutrition, Akershus University Hospital HF, Lørenskog, Norway; 3 Multidisciplinary Laboratory Medicine and Medical Biochemistry, Akershus University Hospital HF, Lørenskog, Norway; 4 Department of Gynecology, Akershus University Hospital HF, Lørenskog, Norway

**Keywords:** Morbid obesity, Bariatric surgery, Pregnancy, Micronutrients, Vitamin K1 deficiency

## Abstract

**Background:**

Maternal obesity is associated with adverse outcome for pregnancy and childbirths. While bariatric surgery may improve fertility and reduce the risk of certain pregnancy-related complications such as hypertension and gestational diabetes mellitus, there is a lack of evidence on the optimal nutritional monitoring and supplementation strategies in pregnancy following bariatric surgery. We aimed to assess the impact of bariatric surgery on micronutrients in post-bariatric pregnancy and possible differences between gastric bypass surgery and sleeve gastrectomy.

**Methods:**

In this prospective case control study, we recruited 204 pregnant women (bariatric surgery *n* = 59 [gastric bypass surgery *n* = 26, sleeve gastrectomy *n* = 31, missing *n* = 2] and controls *n* = 145) from Akershus university hospital in Norway. Women with previous bariatric surgery were consecutively invited to study participation at referral to the clinic for morbid obesity and the controls were recruited from the routine ultrasound screening in gestational week 17–20. A clinical questionnaire was completed and blood samples were drawn at mean gestational week 20.4 (SD 4.5).

**Results:**

The women with bariatric surgery had a higher pre-pregnant BMI than controls (30.8 [SD 6.0] vs. 25.2 [5.4] kg/m2, *p* < 0.001). There were no differences between groups regarding maternal weight gain (bariatric surgery 13.3 kg (9.6) vs. control 14.8 kg (6.5), *p* = 0.228) or development of gestational diabetes (*n* = 3 [5%] vs. *n* = 7 [5%], *p* = 1.000). Mean levels of vitamin K1 was lower after bariatric surgery compared with controls (0.29 [0.35] vs. 0.61 [0.65] ng/mL, *p* < 0.001). Multiadjusted regression analyses revealed an inverse relationship between bariatric surgery and vitamin K1 (B -0.26 ng/mL [95% CI -0.51, -0.04], *p* = 0.047) with a fivefold increased risk of vitamin K1 deficiency in post-bariatric pregnancies compared with controls (OR 5.69 [1.05, 30.77] *p* = 0.044). Compared with sleeve gastrectomy, having a previous gastric bypass surgery was associated with higher risk of vitamin K1 deficiency (OR 17.1 [1.31, 223.3], *p* = 0.030).

**Conclusion:**

Post-bariatric pregnancy is negatively associated with vitamin K1 with a higher risk of vitamin K1 deficiency in pregnancies after gastric bypass surgery compared with after sleeve gastrectomy. Vitamin K1 deficiency in post-bariatric pregnancy have potential risk of hypocoaguble state in mother and child and should be explored in future studies.

## Background

Obesity is common in women of reproductive age, increasing the risk of several complications for mother and child [1, 2]. Maternal metabolism in obesity may reduce the likelihood of successful pregnancy [3]. Moreover, given that weight loss before pregnancy mitigates the adverse outcomes of pregnancy related outcomes from obesity, bariatric surgery in women of reproductive age in increasing [4, 5]. However, although bariatric surgery may reduce the risks of certain obesity related complications in pregnancy, pregnancy after bariatric surgery may carry adverse events such as malnutrition, vitamin deficiencies and inadequate weight gain as well as changes in endocrine and metabolic homeostasis [6–10]. Pregnancy following bariatric surgery has been associated with increased risk of preterm birth, nutritional deficiency and small for gestational age [7, 8, 11–14]. The causality of these effects are not known, but personalized nutritional counseling during post-bariatric pregnancy has been shown to improve nutrient intake of mothers and may contribute to higher weight of offspring [15].

There is a growing body of evidence suggesting that maternal nutrition and lifestyle affect fetal growth and development [16, 17]. Micronutrients are vitamins and minerals that enable the body to produce enzymes, hormones and other substances essential for normal growth and development [18]. Micronutrient deficiencies contribute to poor growth, intellectual impairments and increased risk of morbidity and mortality [19]. Widespread global micronutrient deficiencies exist, with pregnant women and young children at highest risk [19]. Micronutrient interventions such as supplementation of folate to prevent neural tube defects zinc to reduce risk of preterm birth, and iron to reduce the risk of low birthweight are established [20–22]. The micronutritional deficiencies seen after bariatric surgery might be explained by poor dietary pattern in combination with gastrointestinal modification and reduced intestinal transit time [23–27]. Deficiencies of fatty soluble vitamins seem to be particularly prevalent in post-bariatric pregnancies, with potential risks of impaired vision, neuronal disorders, impairment of the immune system and hypocoagulability for mother and child [24, 28–30].

While sleeve gastrectomy is the most common surgical procedure for the treatment of obesity worldwide, there is conflicting evidence on the optimal surgical procedure before subsequent pregnancy [10, 31]. A large registry study showed no difference between gastric bypass and sleeve gastrectomy for preterm birth or small for gestational age [12]. Studies indicates increased risk of prematurity in pregnancy occurring less than 2 years after bariatric surgery [12, 32]. However, other studies have not confirmed increased risks in pregnancies related to time-interval between bariatric surgery and conception [13, 33]. As such, there is an evident knowledge gap on the impact of bariatric surgery on micronutrient status in pregnancies as well as outcomes for mother and child in order to provide optimal obstetric care in this group.

The aim of this study was to assess the impact of bariatric surgery on concentrations of micronutrients in post-bariatric pregnancies compared with non-surgical controls. Specifically, we hypothesized that fatty soluble micronutrients, including vitamin K1, was impaired after bariatric surgery. We also wanted to assess differences in maternal micronutrients concentrations following sleeve gastrectomy versus gastric bariatric surgery.

## Materials and methods

### Design and study population

This observational case control study compared micronutritional status in pregnancy after bariatric surgery with non-surgical controls. Study participants were recruited from Akershus university hospital, between October 18th 2018 and December 9th 2022. Pregnant women with previous bariatric surgery were consecutively invited to study participation at referral to the clinic for morbid obesity and the controls were recruited from the routine ultrasound screening in gestational week 17–20. A total of 59 women with a previous bariatric surgery was included in the study and information on surgical procedure was available for 57 women (gastric bypass surgery *n* = 26 and sleeve gastrectomy *n* = 31). All women with post-bariatric pregnancies were closely monitored individually by a clinical doctor and a registered clinical dietitian focusing on micronutrient status and gestational weight gain. The controls received standard hospital care and dietary advice with additional advice if the blood samples revealed deficiencies.

A total of 204 women were included in this study with 92% of Caucasian ethnicity (*n* = 185). We compared micronutrient status in pregnancy in women with previous bariatric surgery (*n* = 59) to controls (*n* = 145). Women with known intestinal conditions (i.e. known inflammatory bowel disease, uncontrolled coeliac disease) were not included in the study. The study was approved by the Regional Committee for Medical and Health Research Ethics (reference 25829). All study participants provided written informed consent before study commencement, and the study was performed in accordance with the Declaration of Helsinki [34].

### Definitions

The reference intervals for micronutrients in non-pregnant women and the chosen cut-offs defining micronutrient deficiencies in pregnancy are presented in Table 1. We defined micronutrient deficiency according to known physiological changes in blood during pregnancy combined with established reference intervals in a non-pregnant population [30, 35–37]. Time interval between bariatric surgery and conception was categorized into < 18 and ≥ 18 months.

**Table 1:** Non-pregnant reference intervals for the micronutrients and the chosen clinical decision limits defining micronutrient deficiencies in pregnancy [30, 35–37]

Micronutrient	Non-pregnant reference intervals	Clinical decision limits for deficiency in pregnancy
Ferritin (Iron-status)	10–170 µg/L	< 30 µg/L
Magnesium	0.71–0.94 mmol/L	< 0.7 mmol/L
Cobalamin	150–650 pmol/L	< 276 pmol/L
Folate	≥ 7 nmol/L	< 15 nmol/L
Pyridoxal 5-phosphate	15–160 nmol/L	< 15 nmol/L
Thiamine	95–200 nmol/L	< 95 nmol/L
Vitamin A	1.2–3.6 µmol/L	< 1,2 µmol/L
Vitamin D	50–125 nmol/L	< 50 nmol/L
Vitamin E	19–50 µmol/L	< 19 µmol/L
Vitamin K1	0.1–2.2 ng/mL	< 0.1 ng/mL
Zinc	10–18 µmol/L	< 7.6 µmol/L
Selenium	0.6–1.8 µmol/L	< 0.9 µmol/L

### Data collection

Clinical and laboratory data were retrieved at mean gestational week 20.4 (SD 4.5) (Bariatric surgery 23.9 [6.5] vs. controls 19.0 [2.0] weeks, *p* < 0.001). Follow-up blood sample was available in a subgroup of 32 women with post-bariatric pregnancies at mean 30.4 (SD 5.6) gestational week. All patients completed a questionnaire on comorbidities, medications and dietary supplements. Additional information including maximum weight, time of bariatric surgery, type of bariatric surgery was retrieved during the first visit.

### Blood samples and analysis

The blood samples were obtained by venipuncture and collected in Vacuette® tubes. EDTA tubes were used for analysis of hemoglobin, hemoglobin A1c and thiamine (vitamin B1). Lithium heparin gel tubes were used for analysis of zinc and selenium, and serum gel tubes for the remaining analyses. All the blood samples were non-fasting. After blood collection, all tubes were handled according to established procedures. The standard clinical chemistry parameters were analysed at the laboratory at Akershus University Hospital. Hemoglobin was analysed on Sysmex instruments (Sysmex Corporation, Kobe, Japan) and hemoglobin A1c on Tosoh instruments (Tosoh Corporation, Tokyo, Japan). Magnesium and homocysteine were analysed on Vitros 5.1 FS (Ortho Clinical Diagnostics, Raritan, NJ) until May 2021, thereafter on cobas c503 (Roche Diagnostics, Mannheim, Germany). Folate, cobalamin, ferritin and vitamin D were analysed on cobas e801 (Roche Diagnostics). Zinc and selenium were analysed using inductive coupled plasma – mass spectrometry (ICP-MS) and methylmalonic acid (MMA) with a liquid chromatography – mass spectrometry method (LC-MS/MS). Thiamine, pyridoxal 5-phosphate (vitamin B6), vitamin A and vitamin E were analysed at Oslo University Hospital, Aker and vitamin K1 was analysed at Fürst Medical Laboratorium, Oslo, all with chromatographic methods.

### Statistical analysis

We estimated that the prevalence of micronutrient deficiency would be 30% in post-bariatric pregnancies and 5% in controls. To confirm a similar difference with a statistical power of more than 80% and a significance level (α) of 0.05, a total of 200 patients had to be included in the study with a 4:1 ratio of cases vs. controls (40 post-bariatric pregnancies and 160 controls). Proportions are reported as numbers with percent, continuous variables as mean ± standard deviation (SD) as appropriate. Differences between treatment groups were analysed using Pearson’s chi-square test or Fishers exact test for categorical data and Student’s t-test for continuous data. Paired sample t-test was used to assess paired observations of micronutrients in baseline and follow-up blood samples. Skewed distributed data were log-transformed to achieve normal distribution. Correlations between possible confounders and vitamin K1 variables were assessed by Spearman’s correlation (rho). Two-sided P values < 0.05 were considered statistically significant. The Bonferroni Holm correction was applied to mitigate the risk of type 1 statistical error. We used linear regression analyses to explore possible associations between bariatric surgery and vitamin K1 and logistic regression analyses to explore possible associations between bariatric surgery and vitamin K1 deficiency. Possible confounders were identified using a stepwise selection approach in which variables with p-values below 0.10 were included along with clinically significant confounders. Coefficients and odds ratio (OR) from regression analysis are presented with 95% confidence interval (CI). The analyses were performed using IBM SPSS Statistics (version 729.0.0).

## Results

We included 204 women in the study (bariatric surgery *n* = 59 and controls *n* = 145). Data on the specific type of surgical procedure were available for 57 women who had undergone bariatric surgery prior to conception (gastric bypass surgery *n* = 26 and sleeve gastrectomy = 31). The women in the surgical group lost on average 39.0 (16.9) kg from the time of surgery to the start of pregnancy and the time interval from bariatric surgery to pregnancy was mean 63.7 (39.2) months. Patients’ characteristics by surgical status are presented in Table 2.

**Table 2:** Patients’ characteristics by surgical status in post-bariatric pregnancy and in non-surgical controls. Data are presented as mean (SD) or percentages

	Bariatric surgery (*n* = 59)	Control (*n* = 145)	*P*-value
*Maternal*			
Age, yrs	32.1 (5.7)	31.2 (4.2)	0.215
Pre-pregnant BMI, kg/m2	30.8 (6.0)	25.2 (5.4)	< 0.001*
Pre-pregnant obesity (BMI ≥ 30 kg/m2)	30 (52%)	25 (18%)	< 0.001*
Maternal weight gain, kg	13.3 (9.6)	14.8 (6.5)	0.228
Type 2 diabetes	1 (2%)	0 (0%)	0.289
HbA1c, mmol/mol	30.2 (7.1)	31.1 (3.6)	0.234
Gestational diabetes	3 (5%)	7 (5%)	1.000
Sectio	11 (20%)	20 (15%)	0.384
Higher education	24 (43%)	103 (72%)	< 0.001*
Current smoker	5 (9%)	0	0.001*
*Child*			
Gestasjonal age, weeks	38.5 (3.1)	39.3 (2.1)	0.054
Birthweight, g	3363 (624)	3520 (521)	0.081
Preterm birth	3 (6%)	11 (8%)	0.761

* denotes statistically significance after correction for multiple comparisons

The women with bariatric surgery had a higher pre-pregnant body mass index (BMI) compared with controls (30.8 [SD 6.0] vs. 25.2 [5.4] kg/m^2^, *p* < 0.001). There was no difference between groups regarding age (32.1 [5.7] vs. 31.2 [4.2] years, *p* = 0.215), maternal weight gain (13.3 [9.6] vs. 14.8 [6.5] kg, *p* = 0.228), HbA1c (30.2 [7.1] vs. 31.1[3.6] mmol/mol, *p* = 0.234) or development of gestational diabetes (5% vs. 5%, *p* = 1.000). Fewer women with bariatric surgery had completed higher education and more women with bariatric surgery currently smoked compared with controls (24 [43%] vs. 103 [72%], *p* < 0.001 and 5 [9%] vs. 0, *p* = 0.001, respectively. Children of post-bariatric pregnancies had lower gestational age and lower birthweight, however neither reached statistical significance (38.5[3.1] vs. 39.3[2.1] weeks, *p* = 0.054 and 3363 [624] vs. 3520 [521] g, *p* = 0.081, respectively).

**Fig. 1:**
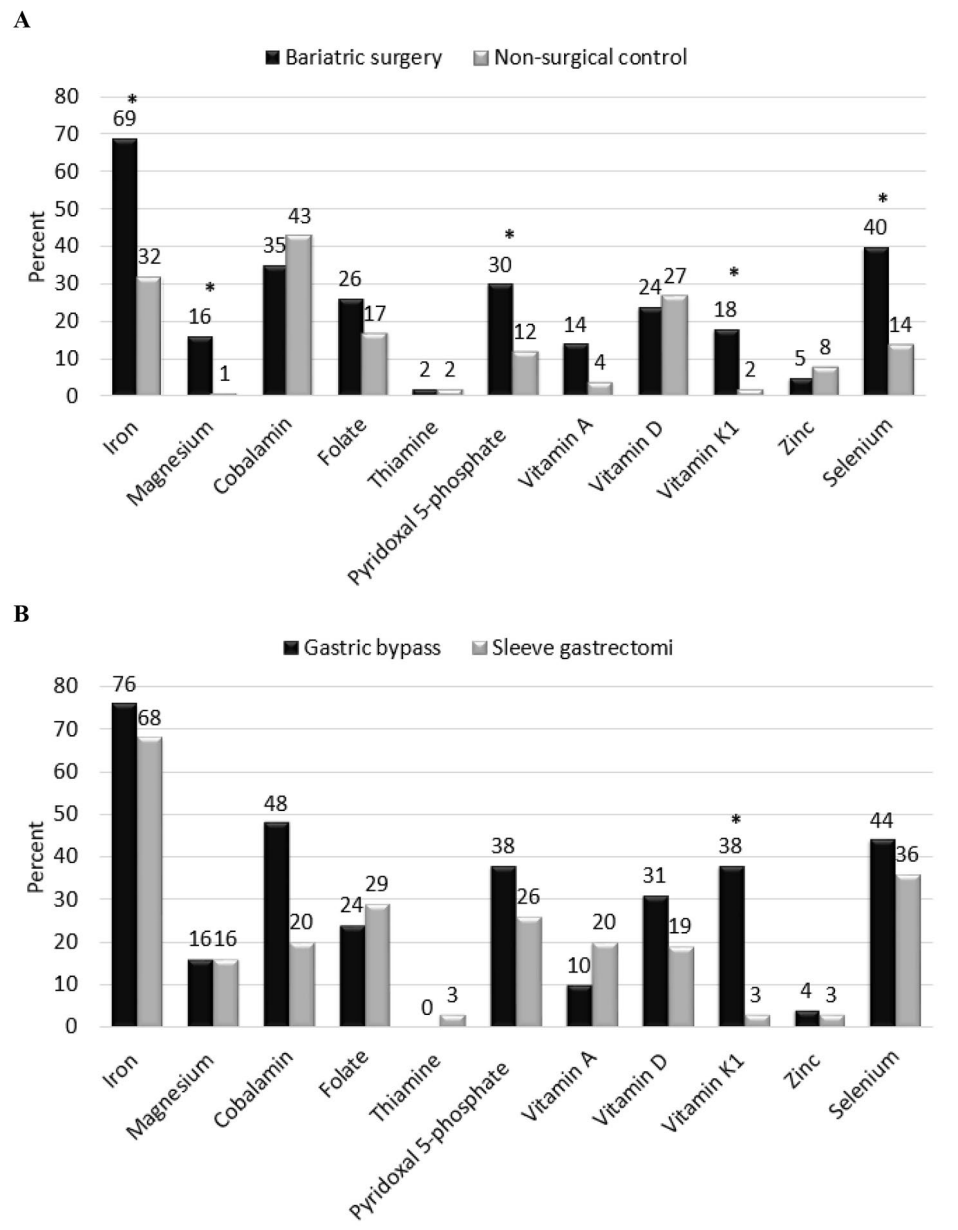
Micronutritional deficiency in pregnancy. **A**: pregnancy following bariatric surgery vs. non-surgical controls. **B**: pregnancy after gastric bypass surgery vs. sleeve gastrectomy. * denotes statistically significance after corrections for multiple comparisons

**Fig. 2:**
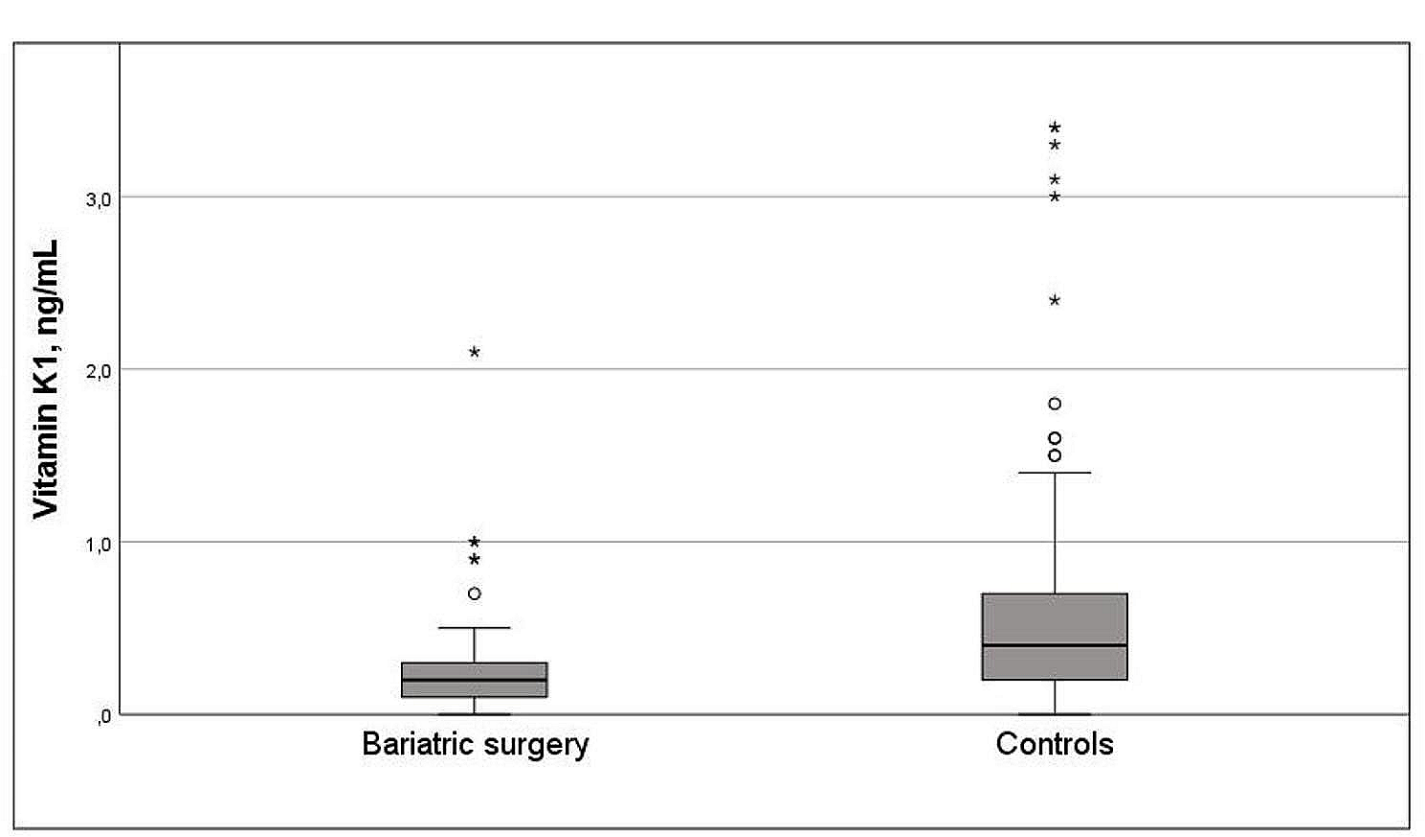
Distribution of vitamin K1 concentrations in women with post-bariatric pregnancies and controls

Dietary supplements and micronutrient status by surgical status are presented in Table 3. Concentrations of ferritin, magnesium, pyridoxal 5-phosphate, vitamin A, E and K1 and selenium were significantly lower post-bariatric pregnancies compared with controls. Using micronutrients as categorical variables (deficiency yes/no) conferred a higher prevalence of micronutrient deficiencies such as iron, magnesium, pyridoxal 5-phosphate, vitamin K1 and selenium in pregnancies after bariatric surgery compared with controls and a higher prevalence of vitamin K1 deficiency after gastric bariatric surgery vs. sleeve gastrectomy (Fig. 1). The distribution of vitamin K1 concentrations in women with post-bariatric pregnancies and controls is presented in Fig. 2. Paired sample t-test showed increased concentrations vitamin K1 in a subgroup of women with post-bariatric pregnancies (0.29 [0.29] ng/mL to 0.64 [0.92] ng/mL, *p* = 0.070).

**Table 3:** Baseline characteristics in pregnancy after bariatric surgery and non-surgical controls. Data are presented as mean (SD) or percentages

	Bariatric surgery (*n* = 59)	Control (*n* = 145)	*P*-value
*Self-reported dietary supplements*:			
Iron	40 (68%)	50 (35%)	< 0.001*
Multivitamin	39 (66%)	67 (46%)	0.013*
Folate	17 (29%)	67 (46%)	0.028*
Cobalamin	33 (56%)	3 (2%)	< 0.001*
Vitamin D	26 (44%)	37 (26%)	0.012*
Calcium	22 (37%)	6 (4%)	< 0.001*
*Micronutrient concentrations*			
Hemoglobin, g/dl	11.7 (1.0)	12.2 (0.9)	0.002*
Ferritin, µg/L	34.9 (43.1)	56.9 (52.7)	< 0.001*
Magnesium, mmol/L	0.76 (0.06)	0.81 (0.05)	< 0.001*
Cobalamin pmol/L	418.3 (299.8)	312.6 (105.7)	0.086
Folate nmol/L	26.6 (14.3)	27.8 (11.8)	0.540
Methylmalonic acid, µmol/L	0.13 (0.1)	0.12 (0.11)	0.514
Homocysteine, µmol/L	6.1 (3.0)	5.9 (4.6)	0.759
Thiamine, nmol/L	145.3 (28.2)	149.0 (25.8)	0.402
Pyridoxal 5-phosphate, nmol/L	23.5 (13.8)	29.4 (17.4)	0.033
Vitamin A, µmol/L	1.50 (0.31)	1.65 (0.31)	0.005
Vitamin D, nmol/L	64.1 (28.0)	61.8 (21.3)	0.519
Vitamin E, µmol/L	35.1 (8.5)	38.0 (6.5)	0.012
Vitamin K1, ng/mL	0.29 (0.35)	0.61 (0.65)	< 0.001*
Zinc, µmol/L	9.67 (1.88)	9.07 (1.19)	0.007
Selenium, µmol/L	0.90 (0.17)	1.03 (0.22)	< 0.001*

* denotes statistically significance after correction for multiple comparisons

**Table 4:** Multiadjusted linear regression analyses with vitamin K1 as dependent variables for pregnant women after bariatric surgery vs. non-surgical controls (A) and after gastric bypass vs. sleeve gastrectomy (B). Effect data are presented as unstandardized beta with 95% confidence interval (CI)

**A:**			
	**Beta**	**95%CI**	*p* **value**
Bariatric surgery	-0.258	-0.513, -0.004	0.047
Vitamin D, nmol/L	0.004	0.000, 0.008	0.033
Vitamin E, µmol/L	0.009	-0.003, 0.021	0.145
Pyridoxal 5-phosphate, nmol/L	0.011	0.005, 0.016	< 0.001
Selenium, µmol/L	0.073	-0.346, 0.491	0.732
Timing of blood sampling, gestational week	0.002	-0.022, 0.026	0.859
Pre-pregnant obesity (BMI ≥ 30 kg/m2)	-0.050	-0.250, 0.151	0.624
Multivitamin supplement	0.055	-0.120, 0.230	0.535
R^2^ = 0.239			
**B:**			
	**Beta**	**95%CI**	*p * **value**
Gastric bypass surgery	0.208	-0.012, 0.428	0.063
Vitamin D, nmol/L	0.005	0.002, 0 008	0.001
Vitamin E, µmol/L	0.006	-0.006, 0.018	0.307
Pyridoxal 5-phosphate, nmol/L	0.003	-0.003, 0.010	0.326
Selenium, µmol/L	0.081	-0.469, 0.630	0.768
Time interval since surgery, months	0.002	-0.001, 0.005	0.126
Pre-pregnant obesity (BMI ≥ 30 kg/m2)	-0.058	-0.260, 0.144	0.565
Multivitamin supplement	0.128	0.0.66, 0.322	0.190
R^2^ = 0.478			

The women with gastric bariatric surgery underwent surgery at a younger age and with a longer time-interval between surgery and conception compared with the women with sleeve gastrectomy (23.5 vs. 27.5 years, *p* = 0.002 and 85 [40] vs. 45 [28] months, p = < 0.001, respectively). One woman (4%) after gastric bariatric surgery and five women (16%) after sleeve gastrectomy, *p* = 0.205 became pregnant < 18 months after surgery. Both surgical groups had lost comparable weight since surgery (gastric bypass surgery 41.4 [17.1] vs. sleeve gastrectomy 37.0 [16.8] kg, *p* = 0.342) and they had comparable pre-pregnant BMI (gastric bypass surgery 31.9 [5.5] vs. sleeve gastrectomy 29.9 [6.4] kg/m^2^, *p* = 0.222). The proportion of women with vitamin K1 deficiency was higher after gastric bariatric surgery compared with sleeve gastrectomy (gastric bypass surgery 9 [38%] vs. 1 [3%], *p* = 0.003 and Fig. 1).

Univariate linear regression analysis showed that bariatric surgery was inversely associated with vitamin K1 levels (B -0.33 [95% CI -0.51, -0.15, *p* < 0.001]. The result remained statistically significant after multivariable adjustments (-0.26 ng/mL [-0.51, -0.04], *p* = 0.047) (Table 4A). In addition, compared with sleeve gastrectomy, gastric bariatric surgery was inversely associated with vitamin K1 in univariate linear regression analysis (0.20 [0.019, 0.387], *p* = 0.031), but not after multivariate adjustment (Table 4B). Using vitamin K1 as a categorical variable (deficiency yes/no), bariatric surgery was associated with a fivefold increased risk of vitamin K1 deficiency compared with controls and that gastric bariatric surgery was associated with higher adjusted risk of vitamin K1 deficiency compared with sleeve gastrectomy (Table 5).

**Table 5:** Multiadjusted logistic regression analyses with vitamin K1 deficiency as dependent variables for pregnant women after bariatric surgery vs. non-surgical controls and in pregnant women after gastric bypass surgery vs. sleeve gastrectomy. Effect data are presented as odds ratio (OR) with 95% confidence interval (CI)

**A:**			
	**OR**	**95%CI**	*p* ** value**
Bariatric surgery	5.69	1.05, 30.77	0.044
Vitamin E, µmol/L	0.84	0.73, 0.96	0.011
Selenium, µmol/L	0.01	0.00, 1.09	0.055
**B:**			
	**OR**	**95%CI**	*p* ** value**
Gastric bypass surgery	17.13	1.31, 223.31	0.030
Vitamin E, µmol/L	0.85	0.71, 1.02	0.077
Selenium, µmol/L	0.00	0.00, 0.41	0.030

## Discussion

In this study, we compare micronutrient concentrations in post-bariatric pregnancy with matched non-surgical controls. The study shows that the concentrations of vitamin K1, magnesium, and selenium were significantly impaired in post-bariatric pregnancies vs. controls. Moreover, our results show that bariatric surgery was consistently associated with vitamin K1 levels, both as a continuous outcome variable and as a categorical variable (vitamin K1 deficiency) in post-bariatric pregnancy compared with controls. Moreover, the associations might be driven by gastric bariatric surgery rather than sleeve gastrectomy. However, the number of pregnant women with vitamin K1 concentration below the lower reference limit was overall small and the confidence intervals were large. Thus, these results should be interpreted with caution.

### Maternal nutrition and micronutrients in pregnancy after bariatric surgery

In pregnancy, there is an increased need for nutrients to support fetal and placental growth and development [20]. A detailed dietary information was not available in this study and we cannot exclude that the women with bariatric surgery had a different nutritional composition compared with controls. In a subgroup of women with post-bariatric pregnancies, an increment in vitamin K was seen. However, the changes did not reach statistical significance. Follow-up blood samples for the controls were not available. A healthy diet after bariatric surgery may differ from the general population in the composition of lean protein, fruits and vegetables and starchy carbohydrates. Nonetheless, the combination of diet, intestinal modifications and increased metabolism in pregnancy might explain the deficiencies in fatty soluble vitamins seen in this study [23–27]. Improved nutrient intake of mothers was seen after personalized nutritional counseling during post-bariatric pregnancy and might contribute to higher birth weight of offspring [15]. Given the complexity and heterogeneity of nutritional status in post-bariatric pregnancies, focusing on sub-groups including pre-gestational nutritional deficiencies, and type of surgery performed is of vital importance. A recent consensus report recommended specialized care in pregnancies after bariatric surgery [38]. There is however a paucity of data to support clinical practice [38, 39]. As such, there is an imperative need to identify pregnancy and trimester specific reference intervals and clinical decision limits in order to help clinical advice on dietary supplement.

Lifelong dietary supplement is recommended after bariatric surgery, however adherence to adequate dietary supplements seems to decrease over time [26, 40, 41]. Our study also confers inadequate use of dietary supplements in pregnancy after bariatric surgery with 30–70% of the women not taking recommended post-bariatric surgery dietary supplements (Table 3). Thus, a need for increased awareness to ensure adequate microntutrional care before, during and after pregnancy is imperative.

### The role of vitamin K1 in pregnancy after bariatric surgery

In line with our results, a systematic review on vitamin K1 concentrations in patients with a history of bariatric surgery reported high risk of vitamin K1 deficiency after bariatric surgery and opted for better monitoring [23]. Our results also cohere with another study of 49 pregnant women with previous bariatric surgery, showing that vitamin K1 concentrations were lower in women with a history of bariatric surgery compared with 27 controls [30]. The increased fat storage in pregnancy may lead to less bioavailability for activation of fatty soluble vitamins [42]. Furthermore, the highly fat-soluble vitamin K1 depend upon conjugated bile salts for adequate absorption. Consequently, reduced stomach acid production, reduced absorption surface and shorter interaction time between conjugated bile salts and vitamin K1 might explain the lower serum concentrations of vitamin K1 after bariatric surgery [43]. Screening for vitamin K1 deficiency is usually recommended after malabsorptive surgical procedures including biliopancreatic diversion with or without duodenal switch [43]. However, restrictive procedures may also cause vitamin deficiencies due to digestive symptoms such as vomiting and food intolerance. Interestingly, lower levels of vitamin K1 were found in the first trimester compared to a control group of women without bariatric surgery [30]. Vomiting and food intolerance may also be the main symptoms of hyperemesis gravidarum, which calls for increased vigilance of vitamin K1 insufficiency in post-bariatric pregnancies in women with symptoms of hyperemesis in pregnancy.

The impact of vitamin K1 deficiency in post-bariatric pregnancies is not clear. Low circulating levels of vitamin K1 might lead to a hypocoaguble state in mother and child [30]. Some cases of neonatal intracranial bleeding have been reported, possible due to vitamin K1 deficiency [44]. Another study reported that obesity had stronger impact on hypercoagulability than pregnancy itself [45]. Nonetheless, insufficient data exist in order to recommend interventions of vitamin K1 deficiency in post-bariatric pregnancy [38]. While optimal monitoring of vitamin K1 during pregnancy following bariatric surgery remains unclear, a major concern is raised about the consistent finding of vitamin K1 deficiency in post-bariatric pregnancy.

### Bariatric surgery before pregnancy: timing and selection of procedure – dose it matter?

Few studies have assessed the impact of different surgical procedures before pregnancy. One study of 119 pregnant women found no effect of maternal weight gain on maternal and perinatal outcome after sleeve gastrectomy [46]. However, the study did not include pregnancies after gastric bariatric surgery for comparison. Another retrospective observational study showed no differences between gastric bariatric surgery and sleeve gastrectomy regarding re-interventions or obstetric outcomes [4]. Conflicting evidence exists on the possible adverse effects of sleeve gastrectomy such as dyspepsia and weight regain as compared with gastric bariatric surgery [47–49]. Our study adds important knowledge about the different surgical procedures, suggesting that gastric bariatric surgery holds greater risk of vitamin K1 deficiency compared with sleeve gastrectomy. The optimal surgical procedure for obesity treatment in women of reproductive age is however not clear and a person-centered approach should be advocated in future guidelines.

The timing of pregnancy after bariatric surgery is moreover under debate. Current recommendations suggest waiting at least 12 months after bariatric surgery before planning a pregnancy [12, 38, 50]. In our study, women with previous sleeve gastrectomy had a shorter time interval between surgery and conception than the women with gastric bariatric surgery. This might reflect that the women who underwent gastric bariatric surgery underwent surgery in an era where gastric bariatric surgery was the most common surgical procedure for weight loss [31]. Interestingly, after adjustments for the time interval since bariatric surgery, gastric bariatric surgery was not associated with vitamin K1 in the linear regression model (Table 4B). Thus, as adherence to dietary supplements is reduced with time after bariatric surgery, we cannot rule out that patient’ adherence to dietary supplement might have influenced the differences between surgical procedure seen in the present study [26, 40, 41]. On the other hand, the time interval between sleeve gastrectomy and conception did not impact maternal and neonatal outcomes in a study of 15 women conceived > 18 months after surgery. The authors concluded that pregnancy after sleeve gastrectomy was overall safe and well-tolerated [33]. Furthermore, a study of 30 women who became pregnant within a mean time of 17 months after gastric bariatric surgery did not appear to confer any serious risks in pregnancy with 90% of the children were born at term with normal birthweight [13]. In our study, only six patients (11%) became pregnant earlier than 18 months after surgery and the study was not designed to assess pregnancy or birth related complications.

### Future implications?

The results of this study underscore the need for increased awareness of nutritional and microntutrional status to ensure adequate obstetric care both before and during post-bariatric pregnancies. Also, this study present important information on adherence to dietary supplement that should be considered in the planning of post-bariatric pregnancies. Moreover, the results of our study rises important questions on the impact of micronutrients deficiencies on future child development.

### Strengths and limitations

The strengths of this study include the prospective design with matched controls. Moreover, definitions for the chosen cut-offs for micronutrient deficiency were chosen according to pregnancy specific reference intervals if established. However, we cannot rule out that the concentrations of the micronutrients change in pregnancy. Thus, the validity of the chosen cut-offs for defining micronutrient deficiency should be assessed in future studies. This study was a small single center study and did not have the statistical power to assess pregnancy related or birth related complications. The majority of the women in this study was Caucasian and the results may not be valid in populations of other ethnicities. The observational design does not provide any causality between variables. Also, we cannot rule out if the difference in gestational week for blood sampling or non-fasting blood samples might have influenced the micronutrient analyses. Finally, use of dietary supplements was self-reported and we cannot be sure that all the study participants adhered with the recommendation.

## Conclusion

This study shows that concentrations of the micronutrients vitamin K1, magnesium, and selenium were significantly impaired in post-bariatric pregnancies compared with controls. We found a negative association between bariatric surgery and vitamin K1 and a higher risk of vitamin K1 deficiency after gastric bariatric surgery compared with sleeve gastrectomy. Vitamin K1 deficiency in post-bariatric pregnancy have potential risk of hypocoaguble state in mother and child and should be assessed in future studies.

## Data Availability

The data used in the present study is not open access or publicly available. The datasets are available from the corresponding author on reasonable request.

## Abbreviations

BMI: Body mass index
OR: Odds ratio
CI: Confidence interval
SD: Standard deviation
